# Supplementary material for: Cardiac Hemorrhage Precedes Hypertension-induced Fibrosis in Plasminogen Activator Inhibitor-1 Deficient Mice
Source: bioRxiv. 2025 Nov 19:2025.11.19.689269. Preprint. [Version 1] doi: 10.1101/2025.11.19.689269 (PMC12667979; doi:10.1101/2025.11.19.689269)
Supplement: Supplement 1 [file NIHPP2025.11.19.689269v1-supplement-1.pdf]

bioRxiv preprint doi: https://doi.org/10.1101/2025.11.19.689269; this version posted November 19, 2025. The copyright holder for this preprint (which was not certified by peer review) is the author/funder, who has granted bioRxiv a license to display the preprint in perpetuity. It is made available under aCC-BY 4.0 International license.

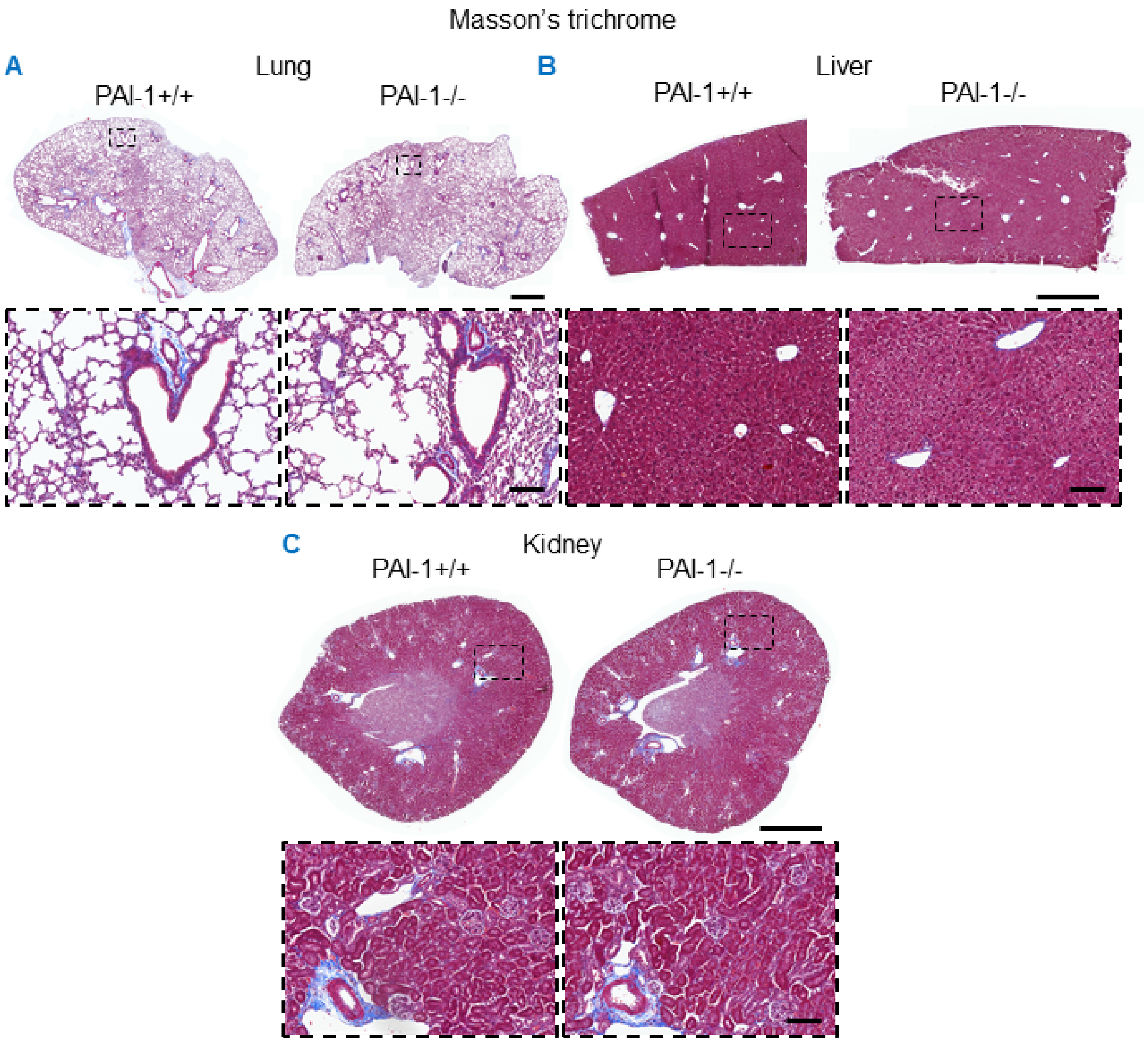

Supplemental Figure 1. PAI-1 deficiency did not overtly alert lung, liver, or kidney fibrosis. (**A-C**) Representative Masson's trichrome staining of left lung (**A**), left liver lobe (**B**), and left kidney sections (**C**) of mice infused with AngII (1,000 ng/kg/min) for 4 weeks. Representative images are from male mice. Scale bars = 1,000 μm and 100 μm for low and high-magnification images, respectively. Abbreviation: AngII: angiotensin II, PAI-1: plasminogen activator inhibitor-1.

bioRxiv preprint doi: https://doi.org/10.1101/2025.11.19.689269; this version posted November 19, 2025. The copyright holder for this preprint (which was not certified by peer review) is the author/funder, who has granted bioRxiv a license to display the preprint in perpetuity. It is made available under aCC-BY 4.0 International license.

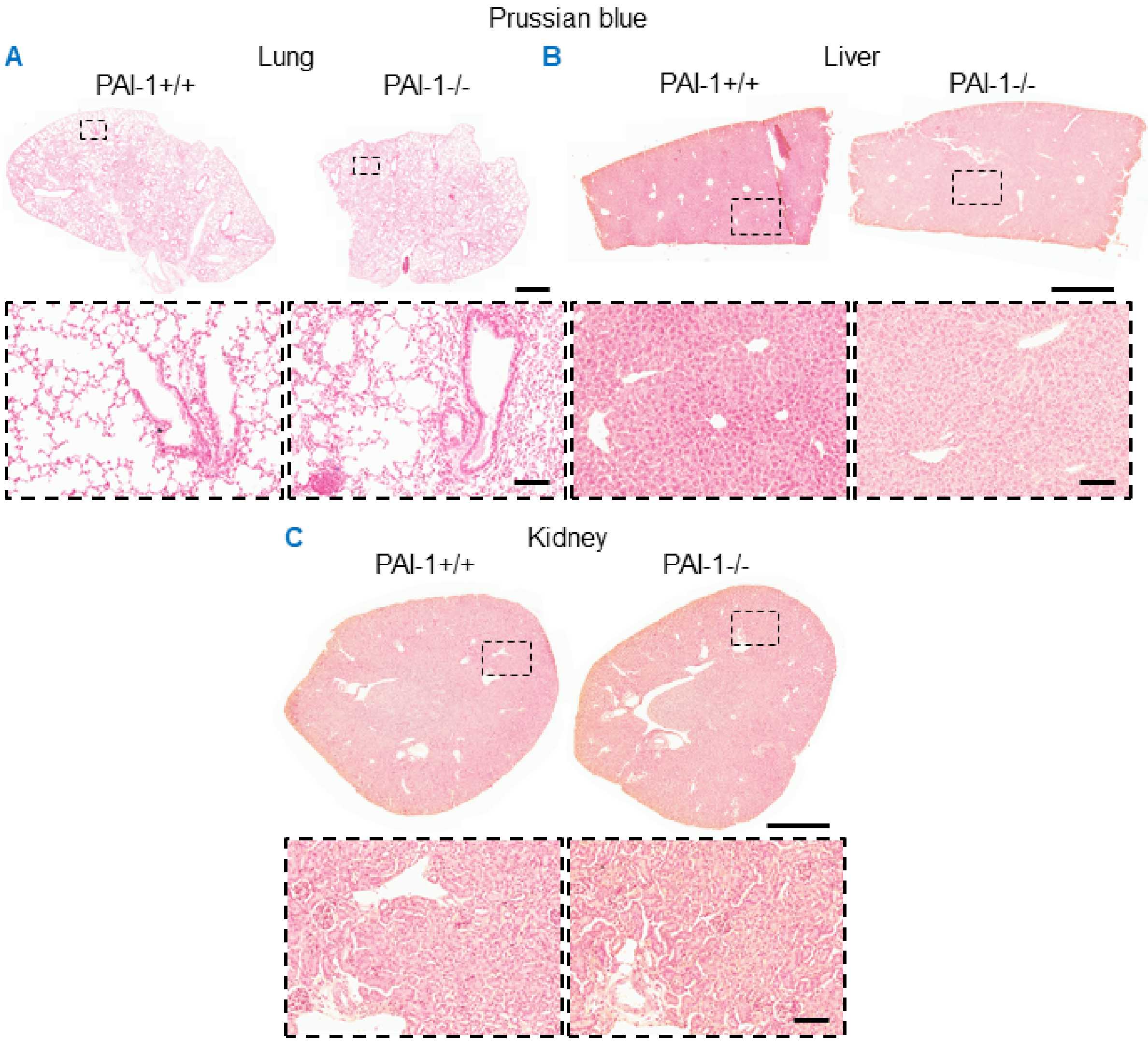

Supplemental Figure 2. PAI-1 deficiency did not detectably alert lung, liver, or kidney ferric iron deposition. (**A-C**) Representative Prussian blue staining of left lung (**A**), left liver lobe (**B**), and left kidney sections (**C**) of mice infused with AngII (1,000 ng/kg/min) for 4 weeks. Representative images are from male mice. Scale bars = 1,000 μm and 100 μm for low and high-magnification images, respectively. Abbreviation: AngII: angiotensin II, PAI-1: plasminogen activator inhibitor-1.

bioRxiv preprint doi: https://doi.org/10.1101/2025.11.19.689269; this version posted November 19, 2025. The copyright holder for this preprint (which was not certified by peer review) is the author/funder, who has granted bioRxiv a license to display the preprint in perpetuity. It is made available under aCC-BY 4.0 International license.

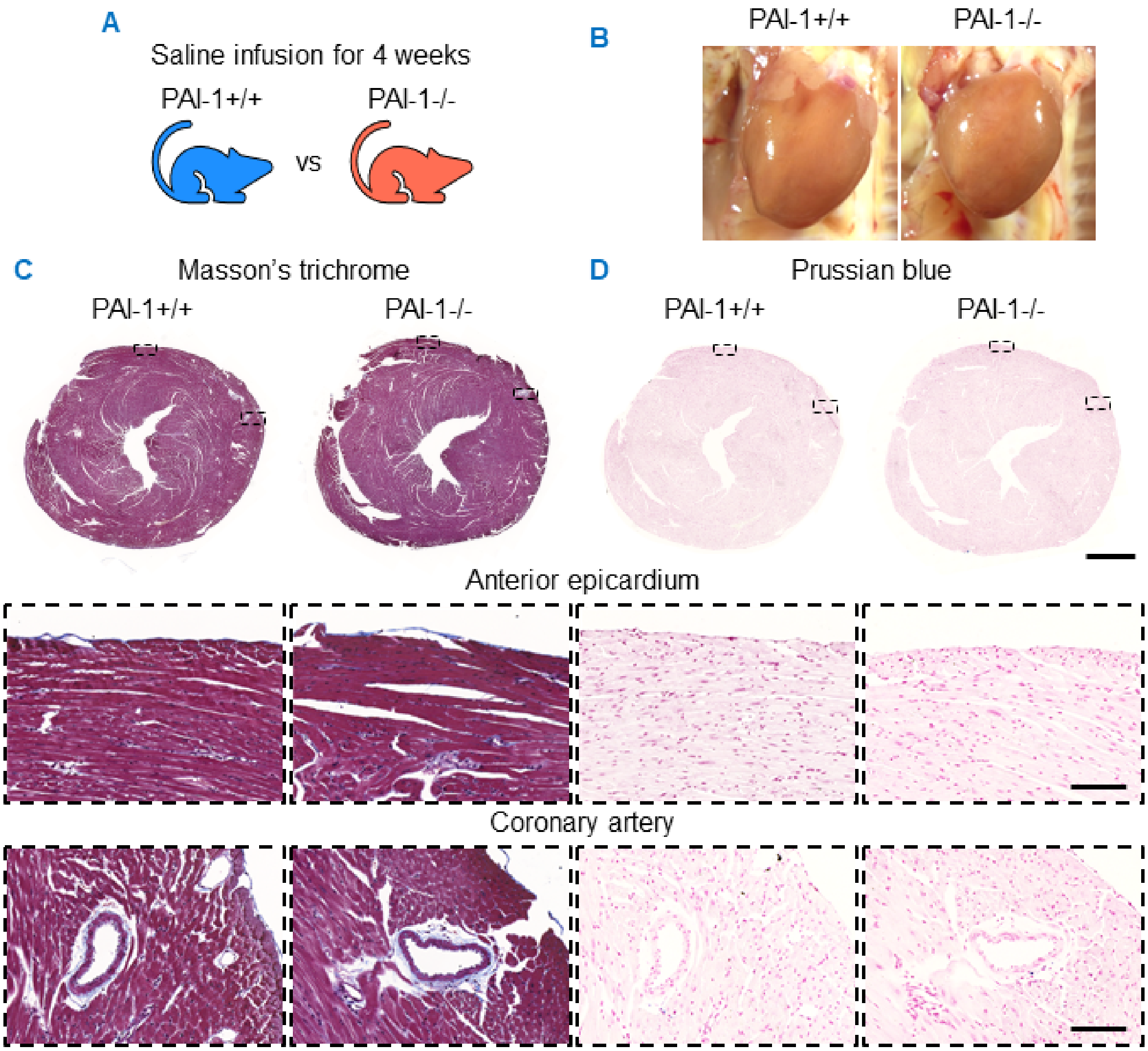

Supplemental Figure 3. PAI-1 deficiency caused minimal cardiac pathology after 4 weeks of saline infusion. (**A**) Male and female PAI-1+/+ and -/- littermates were infused with saline for 4 weeks. (**B**) Representative *in situ* heart images. (**C-D**) Representative Masson's trichrome (**C**) and Prussian blue staining (**D**) of hearts. Representative images are from male mice. Scale bars = 1,000 μm and 100 μm for low and high-magnification images, respectively. Abbreviation: PAI-1: plasminogen activator inhibitor-1.

bioRxiv preprint doi: https://doi.org/10.1101/2025.11.19.689269; this version posted November 19, 2025. The copyright holder for this preprint (which was not certified by peer review) is the author/funder, who has granted bioRxiv a license to display the preprint in perpetuity. It is made available under aCC-BY 4.0 International license.

A

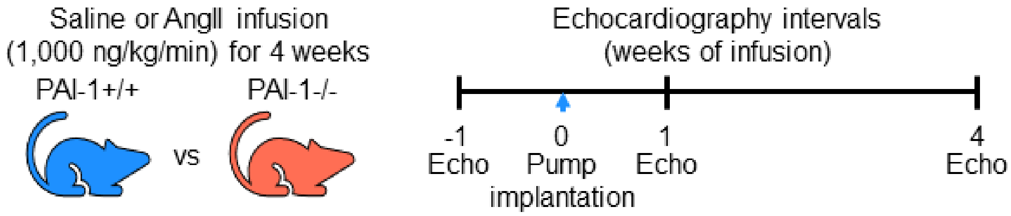

B

Males

Group	Time (weeks)	LVID;d (mm)	LV mass (mg)	LVAW;d (mm)	LVPW;d (mm)	EF (%)	GLS (%)	GCS (%)
PAI-1+/+ saline	-1	3.6 ± 0.2	105 ± 7	0.9 ± 0.05	0.8 ± 0.02	59 ± 3	-21 ± 1	-19 ± 1
	1	4 ± 0.1	117 ± 4	0.9 ± 0.03	0.7 ± 0.03	56 ± 1	-20 ± 2	-22 ± 1
	4	4.1 ± 0.1	130 ± 5	0.9 ± 0.02	0.8 ± 0.04	59 ± 5	-16 ± 1	-22 ± 3
PAI-1-/- saline	-1	3.9 ± 0.2	115 ± 11	0.9 ± 0.07	0.7 ± 0.05	58 ± 7	-20 ± 2	-22 ± 3
	1	3.9 ± 0.1	120 ± 6	0.9 ± 0.03	0.8 ± 0.08	66 ± 2	-18 ± 2	-22 ± 2
	4	3.9 ± 0.1	123 ± 6	0.9 ± 0.05	0.8 ± 0.03	60 ± 3	-16 ± 2	-21 ± 2
PAI-1+/+ AngII	-1	3.8 ± 0.1	108 ± 7	0.9 ± 0.08	0.7 ± 0.03	63 ± 4	-16 ± 1	-22 ± 2
	1	3.9 ± 0.2	149 ± 7	1.1 ± 0.09	0.9 ± 0.03	53 ± 2	-12 ± 2	-16 ± 2
	4	4.1 ± 0.1	148 ± 10	1.1 ± 0.08	0.8 ± 0.06	48 ± 5	-12 ± 2	-17 ± 3
PAI-1-/- AngII	-1	4 ± 0.2	117 ± 4	0.9 ± 0.05	0.7 ± 0.03	57 ± 3	-18 ± 1	-20 ± 2
	1	3.6 ± 0.2	199 ± 25	1.4 ± 0.19	1.1 ± 0.08	55 ± 6	-8 ± 1	-17 ± 2
	4	4.4 ± 0.3	171 ± 20	1.1 ± 0.06	0.8 ± 0.04	48 ± 4	-10 ± 2	-15 ± 1
p values	Group	> 0.99	0.003	0.056	0.009	0.18	< 0.001	0.04
	Time	0.026	< 0.001	0.002	< 0.001	0.11	< 0.001	0.5
	Interaction	0.24	0.071	0.088	0.002	0.25	0.23	0.28

C

Females

Group	Time (weeks)	LVID;d (mm)	LV mass (mg)	LVAW;d (mm)	LVPW;d (mm)	EF (%)	GLS (%)	GCS (%)
PAI-1+/+ saline	-1	3.6 ± 0.2	83 ± 5	0.8 ± 0.05	0.6 ± 0.05	55 ± 4	-16 ± 2	-23 ± 3
	1	3.7 ± 0.2	97 ± 8	0.8 ± 0.03	0.7 ± 0.04	65 ± 5	-19 ± 2	-23 ± 0
	4	3.6 ± 0.1	106 ± 8	0.9 ± 0.04	0.8 ± 0.04	66 ± 2	-19 ± 2	-23 ± 1
PAI-1-/- saline	-1	3.7 ± 0.1	92 ± 6	0.7 ± 0.02	0.7 ± 0.03	54 ± 2	-16 ± 1	-18 ± 2
	1	3.8 ± 0.1	103 ± 6	0.9 ± 0.05	0.7 ± 0.04	56 ± 2	-16 ± 1	-19 ± 1
	4	3.5 ± 0.1	107 ± 5	0.9 ± 0.05	0.8 ± 0.04	66 ± 3	-18 ± 2	-25 ± 3
PAI-1+/+ AngII	-1	3.5 ± 0.1	86 ± 5	0.8 ± 0.04	0.7 ± 0.02	59 ± 3	-17 ± 1	-23 ± 1
	1	3.7 ± 0.1	114 ± 6	0.9 ± 0.05	0.8 ± 0.03	55 ± 2	-16 ± 2	-19 ± 3
	4	3.7 ± 0.1	120 ± 9	1 ± 0.06	0.8 ± 0.04	58 ± 2	-14 ± 2	-18 ± 2
PAI-1-/- AngII	-1	3.4 ± 0.1	77 ± 4	0.7 ± 0.02	0.7 ± 0.03	60 ± 3	-15 ± 1	-18 ± 2
	1	3.3 ± 0.1	136 ± 19	1.2 ± 0.15	1 ± 0.09	59 ± 2	-11 ± 1	-17 ± 1
	4	3.2 ± 0.04	108 ± 5	1 ± 0.07	0.9 ± 0.04	68 ± 4	-12 ± 1	-21 ± 2
p values	Group	0.010	0.61	0.25	0.070	0.17	0.010	0.10
	Time	0.18	< 0.001	< 0.001	< 0.001	0.007	0.62	0.36
	Interaction	0.46	0.16	0.25	0.010	0.25	0.31	0.23

Supplemental Figure 4. PAI-1 deficiency did not alter systolic function within 4 weeks of AngII infusion. (**A**) Male PAI-1+/+ and -/- littermates were infused with saline or AngII (1,000 ng/kg/min) for 4 weeks. Echocardiography was performed 1 week prior to

bioRxiv preprint doi: https://doi.org/10.1101/2025.11.19.689269; this version posted November 19, 2025. The copyright holder for this preprint (which was not certified by peer review) is the author/funder, who has granted bioRxiv a license to display the preprint in perpetuity. It is made available under aCC-BY 4.0 International license.

pump implant (-1) and at 1 and 4 weeks of infusion. (**B-C**) Summary tables of echocardiography parameters in male (**B**) and female mice (**C**). The following transformations were applied prior to analysis: for males: LVPW;d, EF, GLS, and GCS: no transformation; LVID;d: inverse; LV mass and LVAW;d: Box–Cox; for females: LVID;d, GLS, and GCS: no transformation; LV mass: inverse; LVAW;d, LVPW;d, and EF: Box–Cox. Comparisons between groups were made by two-way repeated measures ANOVA. (**B**) *n* = 7 – 8 mice/group. (**C**) *n* = 4 – 10 mice/group. Abbreviations: AngII: angiotensin II, PAI-1: plasminogen activator inhibitor-1, Echo: echocardiography, LVID;d: left ventricular internal diameter; diastole, LV: left ventricle, LVAW;d: left ventricle anterior wall; diastole, LVPW;d left ventricle posterior wall; diastole, EF: ejection fraction, GLS: global longitudinal strain, GCS: global circumferential strain.

bioRxiv preprint doi: https://doi.org/10.1101/2025.11.19.689269; this version posted November 19, 2025. The copyright holder for this preprint (which was not certified by peer review) is the author/funder, who has granted bioRxiv a license to display the preprint in perpetuity. It is made available under aCC-BY 4.0 International license.

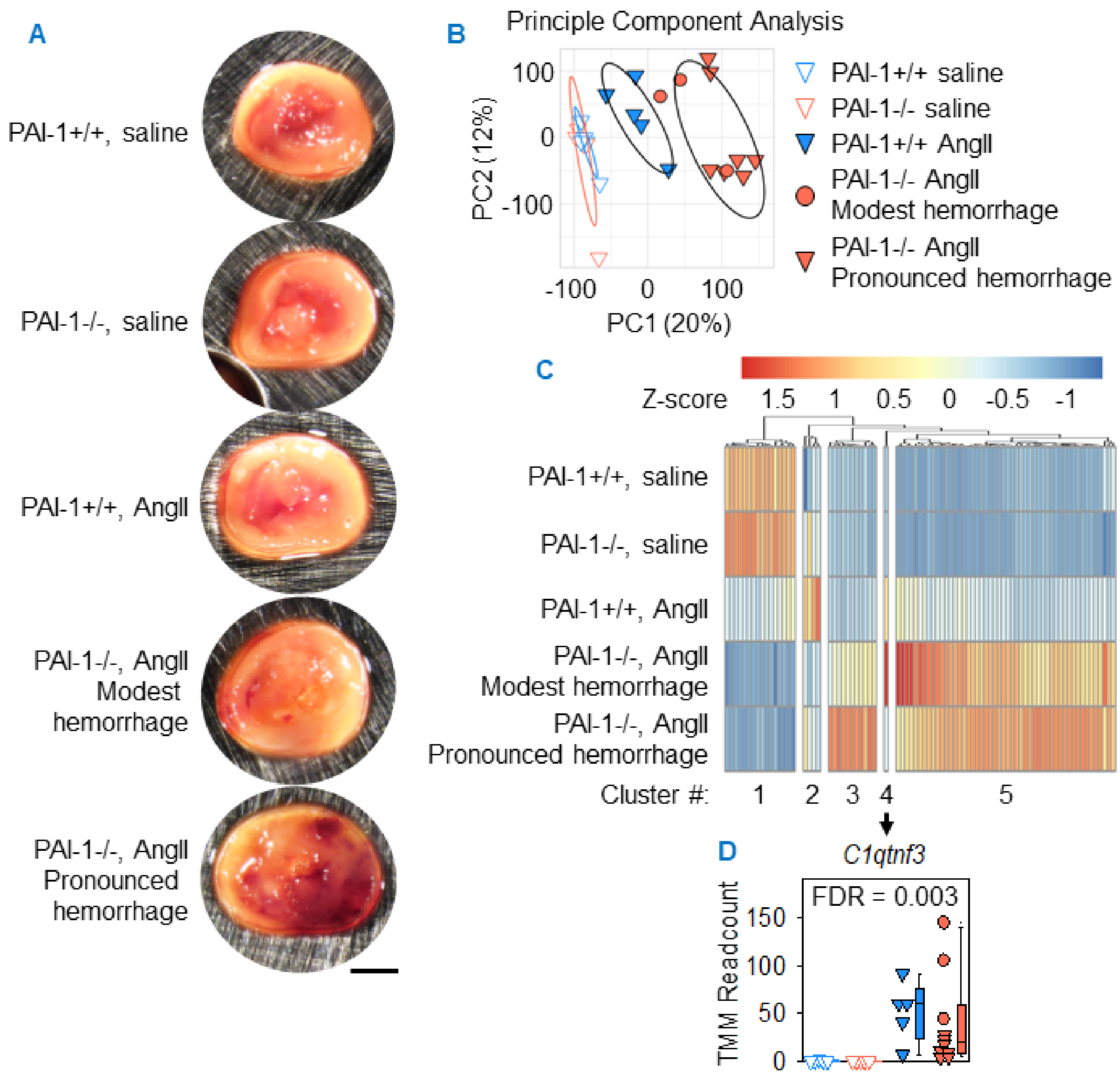

Supplemental Figure 5. PAI-1 deficiency augmented AngII-induced cardiac transcriptomic alterations after 1 week of infusion. Male PAI-1+/+ and -/- littermates were infused with saline or AngII (1,000 ng/kg/min) for 1 week. (**A**) Representative images of mid-ventricular heart sections used for bulk RNA-sequencing. Scale bar = 1,000 µm. (**B**) Principal component analysis of heart transcriptomes. Ellipses represent 75% confidence intervals. (**C**) Z-scored heatmap of all genes differentially expressed by interaction analysis of genotype and infusion controlling for the presence of pronounced hemorrhage. (**D**) Normalized read count expression of *C1qtnf3*, the sole transcript in cluster #4. Legend applies to (**B**) and (**D**). *n* = 5-10 mice/group. Abbreviations: PAI-1: plasminogen activator inhibitor-1, AngII: angiotensin II, PC: principal component, TMM: trimmed mean of M-values, FDR: false discovery rate.

bioRxiv preprint doi: https://doi.org/10.1101/2025.11.19.689269; this version posted November 19, 2025. The copyright holder for this preprint (which was not certified by peer review) is the author/funder, who has granted bioRxiv a license to display the preprint in perpetuity. It is made available under aCC-BY 4.0 International license.

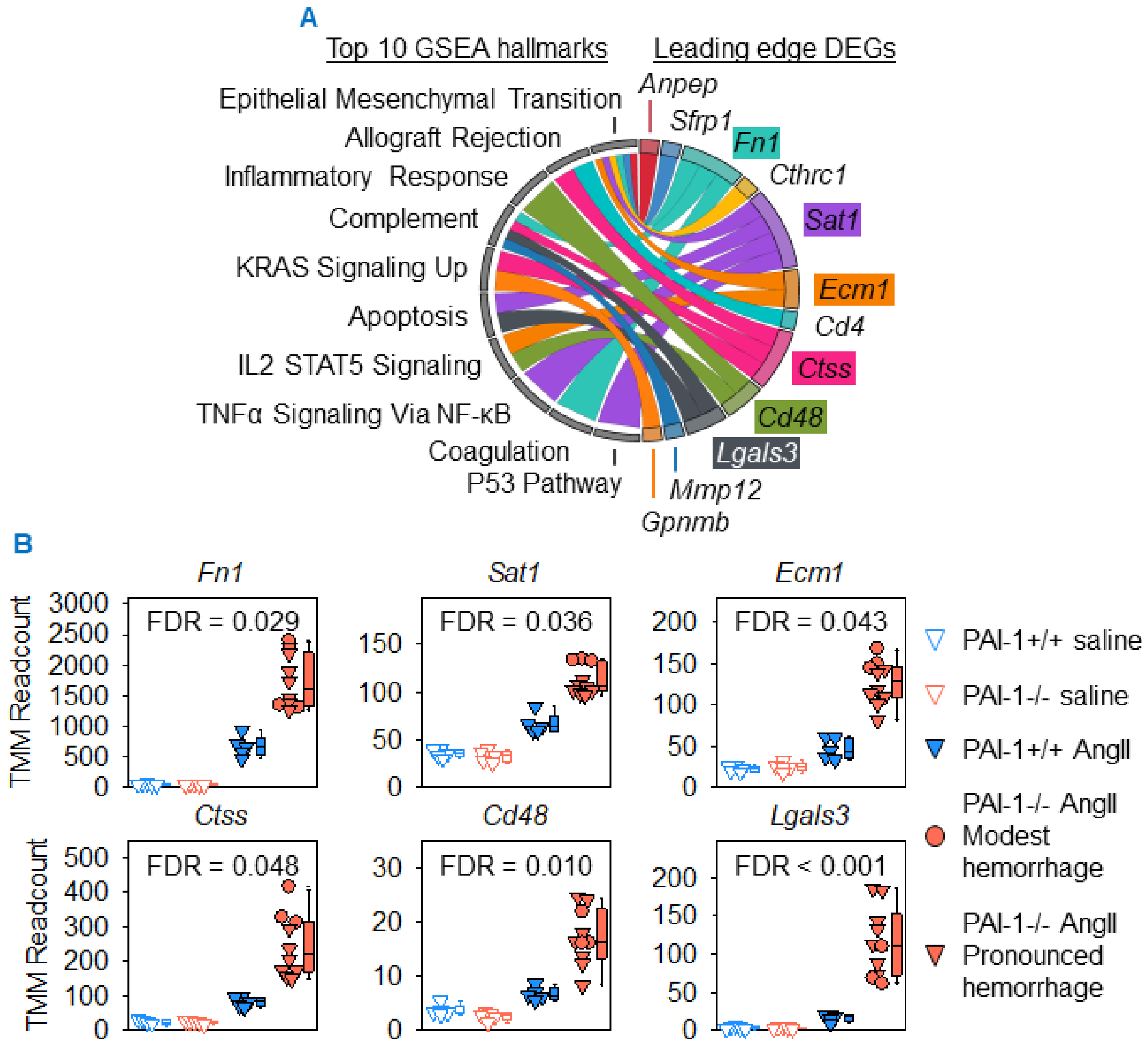

Supplemental Figure 6. PAI-1 deficiency increased transcripts related to proteolysis and fibrosis independently of hemorrhage presence after 1 week of AngII infusion. (**A**) Chord graph representing the top 10 GSEA hallmark gene sets and interaction DEGs present within the leading edge of corresponding Hallmark gene sets. (**B**) Normalized read count expression of interaction DEGs present in the leading edge of at least two top-10 GSEA Hallmark gene sets. *n* = 5-10 mice/group. Abbreviations: AngII: angiotensin II, PAI-1: plasminogen activator inhibitor-1, GSEA: gene set enrichment analysis, DEG: differentially expressed gene, TMM: trimmed mean of M-values, FDR: false discovery rate.

bioRxiv preprint doi: https://doi.org/10.1101/2025.11.19.689269; this version posted November 19, 2025. The copyright holder for this preprint (which was not certified by peer review) is the author/funder, who has granted bioRxiv a license to display the preprint in perpetuity. It is made available under aCC-BY 4.0 International license.

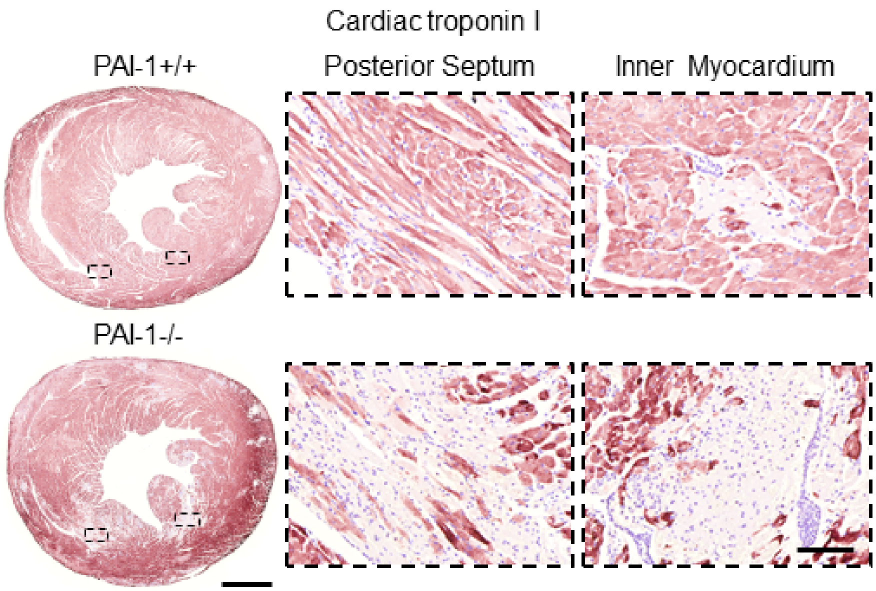

Supplemental Figure 7. PAI-1 deficiency induced cardiomyocyte injury after 1 day of AngII infusion. Male PAI-1+/+ and -/- littermates were infused with AngII (1,000 ng/kg/min) for 1 day. Representative cardiac troponin I immuno-histochemistry in mid-ventricular heart sections. Scale bars = 1,000 μm and 100 μm for low and high-magnification images, respectively. Abbreviations: AngII: angiotensin II, PAI-1: plasminogen activator inhibitor-1.

bioRxiv preprint doi: https://doi.org/10.1101/2025.11.19.689269; this version posted November 19, 2025. The copyright holder for this preprint (which was not certified by peer review) is the author/funder, who has granted bioRxiv a license to display the preprint in perpetuity. It is made available under aCC-BY 4.0 International license.

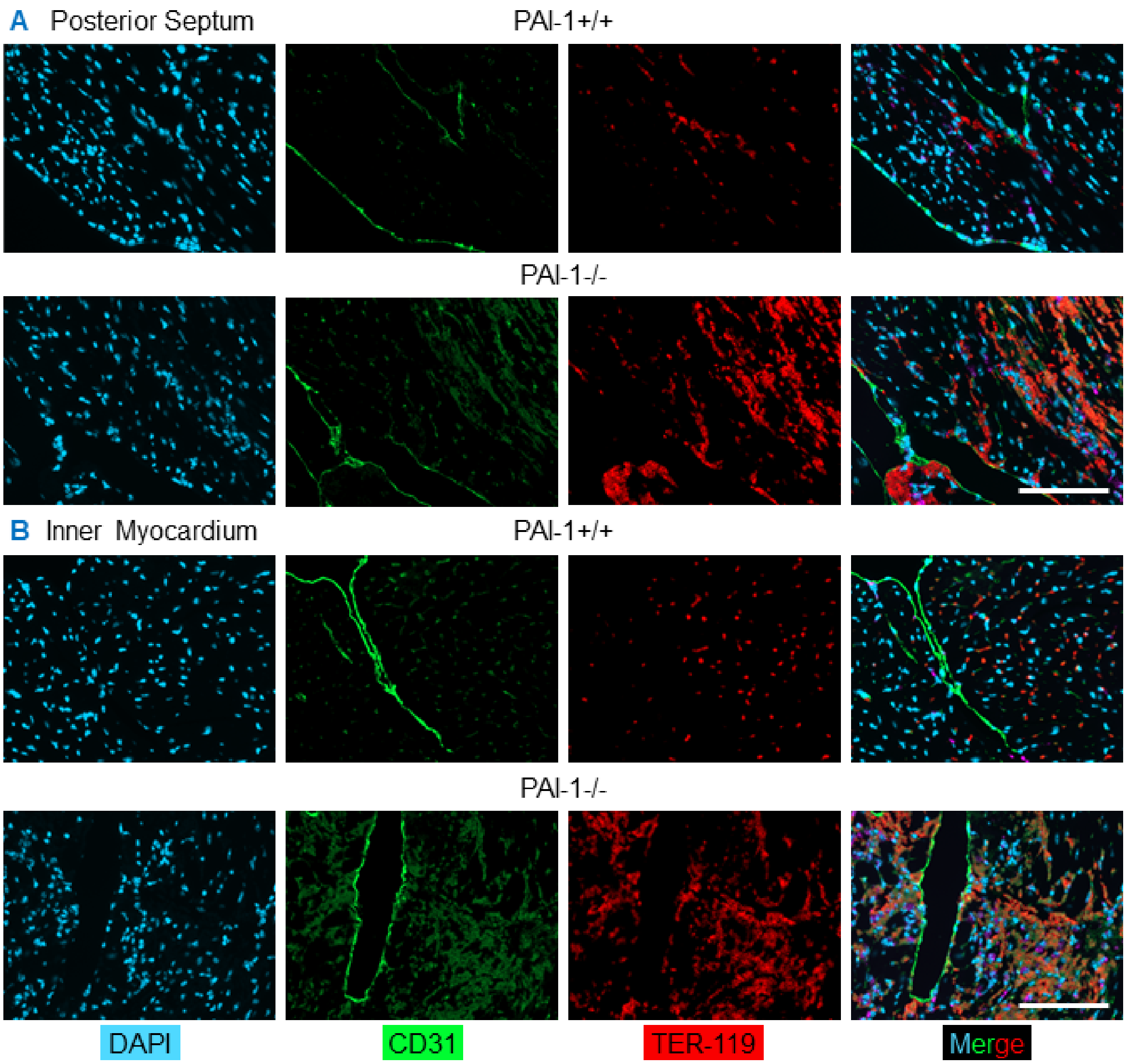

Supplemental Figure 8. PAI-1 deficiency induced erythrocyte extravasation after 1 day of AngII infusion. Male PAI-1+/+ and -/- littermates were infused with AngII (1,000 ng/kg/min) for 1 day. (**A-B**) Representative co-immunofluorescent staining for DAPI, CD31, and TER-119 in (**A**) the posterior septum and (**B**) the inner myocardium of mid-ventricular heart sections. Scale bars = 100 μm. Abbreviations: AngII: angiotensin II, PAI-1: plasminogen activator inhibitor-1, DAPI: 4',6-diamidino-2-phenylindole.

bioRxiv preprint doi: https://doi.org/10.1101/2025.11.19.689269; this version posted November 19, 2025. The copyright holder for this preprint (which was not certified by peer review) is the author/funder, who has granted bioRxiv a license to display the preprint in perpetuity. It is made available under aCC-BY 4.0 International license.

MAJOR RESOURCES TABLES

Primer sequences for genotyping of PAI-1

Gene (Forward / Reverse)	Vendor or Source	Primer Sequence (5'-3')
PAI-1 (forward)	Integrated DNA Technologies	CTGGGCAGTAACCCAAGAGA
PAI-1 (wild type reverse)	Integrated DNA Technologies	GTCGGTCGTCTAGACCCTTG
PAI-1 (mutant reverse)	Integrated DNA Technologies	TGGATGTGGAATGTGTGCGAG

Blocking reagents for immunostaining

Reagent	Vendor	Catalog #
Normal goat serum	Vector laboratories	30024
Normal horse serum	Vector laboratories	30022

Primary antibodies for immunostaining

Antibody	Vendor	Catalog #	Working concentration
Rat anti-TER-119	Thermo Scientific	15-5921-82	0.3 μg/mL
Rabbit anti-cardiac troponin I	abcam	ab47003	1.0 μg/mL
Goat anti-CD31	R&D Systems	AF3628	17.8 μg/mL

Secondary antibodies for immunostaining

Antibody	Vendor	Catalog #	Working concentration
ImmPRESS HRP goat anti-rat IgG	Vector Laboratories	30033	as supplied
ImmPRESS HRP goat anti-rabbit IgG	Vector Laboratories	30125	as supplied
Alexa-647 donkey anti-rat IgG	abcam	ab150155	2.0 μg/mL
Alexa-488 donkey anti-goat IgG	abcam	ab150129	2.0 μg/mL

RGB thresholds for histology and immunostaining quantification

Stain	Feature	Thresholds		
		Red	Green	Blue
Masson's trichrome	Collagen	0–180	0–190	200–255
	Background	220–255	200–255	215–255
Prussian blue	Ferric iron	30–150	40–160	70–240
	Background	250–255	250–255	250–255
TER-119	Erythrocytes	85–200	40–140	10–110
	Background	250–255	250–255	250–255

Figure 1

Inclusion Criteria	Exclusion Criteria
• Male and female<br>• 8-14 weeks of age<br>• PAI-1+/+ and PAI-1-/-	• Death prior to endpoint<br>• Euthanasia prior to endpoint for humane reasons as based on IACUC regulations<br>• Heterozygous genotype determined at endpoint verification

bioRxiv preprint doi: https://doi.org/10.1101/2025.11.19.689269; this version posted November 19, 2025. The copyright holder for this preprint (which was not certified by peer review) is the author/funder, who has granted bioRxiv a license to display the preprint in perpetuity. It is made available under aCC-BY 4.0 International license.

	• Plasma renin concentration (PRC) > 10 ng/ml/hour at endpoint				
**Groups**	**Sex**	**Age (weeks)**	**Number (prior to experiment)**	**Number (termination)**	**Exclusions**
PAI-1+/+, AngII for 4 weeks	M	8-14	14	12	(*n* = 2) death prior to endpoint
PAI-1-/-, AngII for 4 weeks	M	8-14	10	9	(*n* = 1) PRC > 10 ng/ml/hour at endpoint
PAI-1+/+, AngII for 4 weeks	F	8-14	11	11	none
PAI-1-/-, AngII for 4 weeks	F	8-14	22	16	(*n* = 2) heterozygous genotype at endpoint; (*n* = 4) PRC > 10 ng/ml/hour at endpoint

Figure 2

**Inclusion/Exclusion Criteria**					
Inclusion and exclusion criteria are identical to those in Figure 1, with the following additional inclusion criteria:<br>• Random selection within groups when no gross cardiac pathology is present<br>• Selection representative of the full range of observed gross cardiac pathology, when present					
**Groups**	**Sex**	**Age (weeks)**	**Number (termination)**	**Number (histology)**	**Inclusion**
PAI-1+/+, AngII for 4 weeks	M	8-14	12	8	Random selection
PAI-1-/-, AngII for 4 weeks	M	8-14	9	7	Full range of gross pathology
PAI-1+/+, AngII for 4 weeks	F	8-14	11	8	Random selection
PAI-1-/-, AngII for 4 weeks	F	8-14	16	9	Full range of gross pathology

Refer to Figure 1 table for the number of mice prior to experiment initiation and for exclusions.

bioRxiv preprint doi: https://doi.org/10.1101/2025.11.19.689269; this version posted November 19, 2025. The copyright holder for this preprint (which was not certified by peer review) is the author/funder, who has granted bioRxiv a license to display the preprint in perpetuity. It is made available under aCC-BY 4.0 International license.

Figure 3

Inclusion Criteria	Exclusion Criteria
• Male and female<br>• 10-12 weeks of age<br>• PAI-1+/+ and PAI-1-/-<br>• Random selection within groups when no gross cardiac pathology is present<br>• Selection representative of the full range of observed gross cardiac pathology, when present	• Death prior to endpoint<br>• Euthanasia prior to endpoint for humane reasons as based on IACUC regulations<br>• Heterozygous genotype determined at endpoint verification

Groups	Sex	Age (weeks)	Number (prior to experiment)	Number (histology)	Inclusion / Exclusion
PAI-1+/+, NE for 4 weeks	M	11-12	7	5	Exclusion: (n = 1) heterozygous genotype at endpoint; (n = 1) death prior to endpoint<br>Inclusion: random selection
PAI-1-/-, NE for 4 weeks	M	11-12	10	8	Exclusion: none<br>Inclusion: full range of gross pathology
PAI-1+/+, NE for 4 weeks	F	11-12	9	6	Exclusion: none<br>Inclusion: random selection
PAI-1-/-, NE for 4 weeks	F	11-12	10	5	Exclusion: none<br>Inclusion: full range of gross pathology

bioRxiv preprint doi: https://doi.org/10.1101/2025.11.19.689269; this version posted November 19, 2025. The copyright holder for this preprint (which was not certified by peer review) is the author/funder, who has granted bioRxiv a license to display the preprint in perpetuity. It is made available under aCC-BY 4.0 International license.

Figure 4

Inclusion Criteria	Exclusion Criteria
• Male and female<br>• 10-12 weeks of age<br>• PAI-1+/+ and PAI-1-/-<br>• Random selection within groups when no gross cardiac pathology is present<br>• Selection representative of the full range of observed gross cardiac pathology, when present	• Death prior to endpoint<br>• Euthanasia prior to endpoint for humane reasons as based on IACUC regulations<br>• Heterozygous genotype determined at endpoint verification<br>• Plasma renin concentration (PRC) > 10 ng/ml/hour at endpoint

**Groups**	**Sex**	**Age (weeks)**	**Number (prior to experiment)**	**Number (histology)**	**Inclusion / Exclusion**
PAI-1+/+, AngII for 1 week	M	11-12	12	6	Exclusion: (*n* = 2) heterozygous genotype at endpoint<br>Inclusion: random selection
PAI-1-/-, AngII for 1 week	M	11-12	15	5	Exclusion: (*n* = 3) heterozygous genotype at endpoint; (*n* = 3) death prior to endpoint; (*n* = 3) PRC > 10 ng/ml/hour at endpoint<br>Inclusion: full range of gross pathology
PAI-1+/+, AngII for 1 week	F	11-12	18	7	Exclusion: (*n* = 2) heterozygous genotype at endpoint<br>Inclusion: random selection
PAI-1-/-, AngII for 1 week	F	11-12	16	6	Exclusion: (*n* = 1) heterozygous genotype at endpoint; (*n* = 1) death prior to endpoint<br>Inclusion: full range of gross pathology

bioRxiv preprint doi: https://doi.org/10.1101/2025.11.19.689269; this version posted November 19, 2025. The copyright holder for this preprint (which was not certified by peer review) is the author/funder, who has granted bioRxiv a license to display the preprint in perpetuity. It is made available under aCC-BY 4.0 International license.

Figure 5

Inclusion Criteria	Exclusion Criteria
• Male and female<br>• 10-12 weeks of age<br>• PAI-1+/+ and PAI-1-/-<br>• Random selection within groups when no gross cardiac pathology is present<br>• Selection representative of the full range of observed gross cardiac pathology, when present	• Death prior to endpoint<br>• Euthanasia prior to endpoint for humane reasons as based on IACUC regulations<br>• Heterozygous genotype determined at endpoint verification

Groups	Sex	Age (weeks)	Number (prior to experiment)	Number (histology)	Inclusion / Exclusion
PAI-1+/+, NE for 1 week	M	11-12	10	6	Exclusion: (*n* = 1) heterozygous genotype at endpoint<br>Inclusion: random selection
PAI-1-/-, NE for 1 week	M	11-12	9	6	Exclusion: none<br>Inclusion: full range of gross pathology
PAI-1+/+, NE for 1 week	F	11-12	5	5	Exclusion: none<br>Inclusion: all
PAI-1-/-, NE for 1 week	F	11-12	10	6	Exclusion: none<br>Inclusion: full range of gross pathology

Figure 6

Inclusion Criteria	Exclusion Criteria
• Male and female<br>• 10-12 weeks of age<br>• PAI-1+/+ and PAI-1-/-	• Death prior to endpoint<br>• Euthanasia prior to endpoint for humane reasons as based on IACUC regulations<br>• Heterozygous genotype determined at endpoint verification

Groups	Sex	Age (weeks)	Number (prior to experiment)	Number (termination)	Exclusions
PAI-1+/+, AngII for 1 day	M	9-12	6	4	(*n* = 2) heterozygous genotype at endpoint
PAI-1-/-, AngII for 1 day	M	9-12	7	6	(*n* = 1) heterozygous genotype at endpoint

bioRxiv preprint doi: https://doi.org/10.1101/2025.11.19.689269; this version posted November 19, 2025. The copyright holder for this preprint (which was not certified by peer review) is the author/funder, who has granted bioRxiv a license to display the preprint in perpetuity. It is made available under aCC-BY 4.0 International license.

Supplemental Figure 4

Inclusion Criteria	Exclusion Criteria
• Male and female<br>• 10-12 weeks of age<br>• PAI-1+/+ and PAI-1-/-	• Death prior to endpoint<br>• Euthanasia prior to endpoint for humane reasons as based on IACUC regulations<br>• Heterozygous genotype determined at endpoint verification

Groups	Sex	Age (weeks)	Number (prior to experiment)	Number (termination)	Exclusions
PAI-1+/+, saline for 4 weeks	M	10-12	8	7	(*n* = 1) heterozygous genotype at endpoint
PAI-1-/-, saline for 4 weeks	M	10-12	9	8	(*n* = 1) death prior to endpoint
PAI-1+/+, AngII for 4 weeks	M	10-12	10	8	(*n* = 1) heterozygous genotype at endpoint; (*n* = 1) death prior to endpoint
PAI-1-/-, AngII for 4 weeks	M	10-12	11	7	(*n* = 1) heterozygous genotype at endpoint; (*n* = 3) death prior to endpoint
PAI-1+/+, saline for 4 weeks	F	10-12	6	4	(*n* = 2) heterozygous genotype at endpoint
PAI-1-/-, saline for 4 weeks	F	10-12	11	10	(*n* = 1) heterozygous genotype at endpoint
PAI-1+/+, AngII for 4 weeks	F	10-12	7	6	(*n* = 1) death prior to endpoint
PAI-1-/-, AngII for 4 weeks	F	10-12	11	9	(*n* = 1) heterozygous genotype at endpoint; (*n* = 1) death prior to endpoint

bioRxiv preprint doi: https://doi.org/10.1101/2025.11.19.689269; this version posted November 19, 2025. The copyright holder for this preprint (which was not certified by peer review) is the author/funder, who has granted bioRxiv a license to display the preprint in perpetuity. It is made available under aCC-BY 4.0 International license.

Supplemental Figure 5-6

**Inclusion Criteria**	**Exclusion Criteria**
• Male and female<br>• 10-12 weeks of age<br>• PAI-1+/+ and PAI-1-/-<br>• Within AngII-infused PAI-1-/-: visible hemorrhage < 5% and ≥ 5% of tissue area = modest and pronounced hemorrhage, respectively	• Death prior to endpoint<br>• Euthanasia prior to endpoint for humane reasons as based on IACUC regulations<br>• Heterozygous genotype determined at endpoint verification<br>• RNA integrity number (RIN) < 6.8

**Groups**	**Sex**	**Age (weeks)**	**Number (prior to experiment)**	**Number (termination)**	**Exclusion**
PAI-1+/+, saline for 1 week	M	8-12	5	5	none
PAI-1-/-, saline for 1 week	M	8-12	6	5	(*n* = 1) RIN < 6.8
PAI-1+/+, AngII for 1 week	M	8-12	5	5	none
PAI-1-/-, AngII for 1 week	M	8-12	11	10	(*n* = 1) death prior to endpoint

bioRxiv preprint doi: https://doi.org/10.1101/2025.11.19.689269; this version posted November 19, 2025. The copyright holder for this preprint (which was not certified by peer review) is the author/funder, who has granted bioRxiv a license to display the preprint in perpetuity. It is made available under aCC-BY 4.0 International license.

ARRIVE Guidelines Checklists

Item	Application
Ethics	Approved by the University of Kentucky IACUC (2018-2967)
Sex	For 1 and 4-week infusion pathological studies, both male and female animals were examined, and similar findings were reported for both sexes. Since no apparent sexual dimorphism was observed in AngII or NE-induced pathology, only male mice were examined for the 1-week bulk RNA sequencing and 1-day AngII infusion studies.
Inclusion / Exclusion criteria	Criteria were determined *a priori* and are listed for individual experiments within the Major Resources Tables.
Sample size	Described in each figure legend.
Sample size calculation	Figure 1: n = 9/group were calculated to detect a 0.3 ± 0.2 mm (mean ± SD) difference in aortic diameter (t test, α error = 0.05 and power = 0.8).<br>Supplemental Figure 5: n = 9/group were calculated to detect a 20 ± 10% (mean ± SD) difference in ejection fraction (ANOVA, α error = 0.05 and power = 0.8).<br>Supplemental Figure 6: n = 7/group were calculated to detect a 6 ± 3.6% (mean ± SD) difference in cardiac collagen staining (t test, α error = 0.05 and power = 0.8).<br>Sample size calculations were not performed for the remaining figures.
Endpoint	Figure 1: ascending aortic diameters.<br>Figures 2-3: cardiac collagen and ferric iron.<br>Figures 4-5: cardiac collagen and TER-119.<br>Figure 6: cardiac TER-119.<br>Supplemental Figure 5: ejection fraction.
Randomization	Mice were randomly assigned to cages at weaning by sex. After stratifying by age, mice were randomly assigned to study groups. Mice were terminated without predetermined order.
Blinding	Genotyping, measurement of ultrasound images and *in situ* aortas, and quantification of histology were performed by an investigator blinded to the study group information. RNA sequencing library generation was performed by a 3rd party blinded to the study group information. All experimental data were verified by an independent investigator blinded to the study group information.
Statistical analysis	SigmaPlot (v15.0, SYSTAT Software Inc.) or R (v4.5.1).
Statistical method	Described in each figure legend.
Data availability	RNA sequencing data (raw FASTQ and aligned data) will be made publicly available at the Gene Expression Omnibus (GEO) repository. Numerical data that support the findings of this study are provided in the Supporting Data Values file. The data that support the findings of this study are also available from the corresponding authors on reasonable request.
